# Attitudes Underlying Corneal Donation in a Group of Trainee Allied Health Professionals

**DOI:** 10.1371/journal.pone.0053538

**Published:** 2012-12-31

**Authors:** Donal McGlade, Carol McClenahan, Barbara Pierscionek

**Affiliations:** 1 School of Biomedical Science, University of Ulster, Coleraine, Northern Ireland; 2 School of Psychology, University of Ulster, Coleraine, Northern Ireland; 3 Faculty of Science, Engineering and Computing, Kingston University London, Kingston upon Thames, Surrey, England; University of Colorado, United States of America

## Abstract

**Background:**

The focus of this study was to investigate factors that may influence personal willingness to register consent to donate corneal tissue upon death using the theory of planned behaviour in a relatively ethnically homogenous group of trainee allied health professionals. The attainment of this knowledge will be of paramount importance in relation to potential interventions that are designed to change donation-related behaviour.

**Methods:**

A questionnaire-based study was undertaken with 92 pre-registration nurses (mean age 24.0 years (standard deviation ±5.6 years); female:male  = 89:3) enrolled at a University in Northern Ireland. Intention to register consent to donate corneal tissue upon death was assessed using both direct and belief-based measures found in the theory of planned behaviour. Descriptive statistics were used to assess demographic information, with correlation and regression analyses being used to identify factors influencing intentions.

**Results:**

The majority of participants were religious (94.6%, n = 87) and mostly Protestant (58.7%, n = 54) or Catholic (35.9%, n = 33). Generally speaking, the theory of planned behaviour accounted for 84% of the variance in intention to register consent. In relation to the constructs found in the theory of planned behaviour, attitude was found to be the strongest predictor of intention to register consent, with subjective norm being the second strongest predictor. Perceived behavioural control did not significantly predict intention to register consent.

**Conclusions:**

The theory of planned behaviour has allowed an understanding of the factors that influence the personal intentions of a group of future allied health professionals from the same ethnic group to register consent to donate their corneal tissue.

## Introduction

Despite the advancements made in transplantation medicine, one of the major factors that limits and hinders transplantation in the United Kingdom is the shortage of available donor tissues [1]. Although corneal transplantation can help improve and restore sight to those with poor visual function, it has been shown that 10% of people on the national Organ Donor Register are unwilling to donate their corneae [2]. Unfortunately this level of unwillingness has demonstrated a rise of 3% from the previous year [3]. A recent analysis of data in the United Kingdom has shown that significant regional differences exist, especially in relation to the donation of corneal tissue [4]. More importantly, it has been found that Northern Ireland possesses the lowest rate of corneal donation across the United Kingdom for the majority of years examined [4]. While it has been suggested that unwillingness to donate may be based on the individual's attitude, social structures, cultural practices and religious beliefs [4] conclusive evidence has yet to be found.

Several attempts have been made to determine the relationship between attitude and behaviour and how this may influence the decision making process of organ donation [5]–[10]. However, a significant gap in knowledge exists with regards to the opinions and attitudes of nurses, who are the allied health professionals most frequently involved in donor tissue collection [11]. It is notable that whilst nurses play a leading role in the donation and transplantation system [11], the emergence of evidence suggests that this group of health professionals commonly exhibit concerns about their lack of knowledge of organ donation [12]–[18]. Due to the existence of these gaps in knowledge [12]–[18] and the low levels of provisions afforded to health professionals to undertake appropriate training [19]–[20]; health professionals' opinions and practices will therefore more likely be based on a personal locus of control. This perspective may create conflict for the health professional and affect their ability to engage in pro-social behaviours [21]–[28].

This paper presents the factors that influence personal willingness to register consent to donate corneal tissue upon death among a group of trainee allied health professionals based in Northern Ireland. The study focussed on pre-registered nurses at the same stage of training, mostly within a similar age range and in a part of the UK where ethnic homogeneity is high. Participants had not undergone any teaching on organ donation and transplantation and none had worked in this area. The level of variance in knowledge is therefore low. The study used a well-established theoretical framework [29] known as the theory of planned behaviour. The theory of planned behaviour takes into consideration salient beliefs about the likely consequences of the behavior (behavioural beliefs), beliefs about the expectations of others (normative beliefs) and beliefs about those factors that may encourage or prevent performance of the behavior (control beliefs) [30]. Behavioural beliefs produce either a favourable or unfavourable attitude towards the behaviour, normative beliefs result in perceived social pressure or subjective norm to perform or refrain from the behaviour; with control beliefs resulting in a perceived behavioural control with regards to the level of ease or difficulty in performing the behaviour [30]. The theory of planned behaviour proposes that an intention to perform a behaviour will be directly influenced by the individual's attitude, subjective norm and perceived behavioural control [30]. The theory of planned behaviour has been applied to a wide range of health and health-related behaviours, including alcohol consumption [31], food consumption [32], smoking [33] and blood donation [34].

## Methods

### Participants and procedures

The study was designed as a questionnaire-based analysis that received permission from the University of Ulster Research Ethics Committee. A total of 92 students were recruited using convenience sampling from the School of Nursing at the University of Ulster, Northern Ireland. All participants were pre-registered nursing students undertaking a full-time degree course leading to the award of BSc. (Hons). The study was conducted during January 2012. Participation in the study was voluntary and without any form of compensation.

### The questionnaire

To ensure consistency was maintained throughout, a vignette was provided at the beginning of the questionnaire that informed participants about the nature of the study and the behaviour under investigation. The questionnaire consisted of two defined sections: demographics and theory of planned behaviour-based items. Demographical items consisted of gender, age and religion; the theory of planned behaviour section was developed into direct and belief-based items as recommended in a previous study [35]. Participants completed items assessing their intention, attitude, subjective norms and perceived control that were based on findings from the literature [7], [34], [36]–[38]. An elicitation study, conducted in accordance to recommendations [30], was used to construct the belief-based items. The questionnaires were self-explanatory and required participants to respond using a seven-point forced-choice Likert-type scale.

### Measures contained within the questionnaire

#### Attitude

Six items in total were included to assess direct attitude (e.g. For me to register my consent to donate my eyes is good/bad) and had a high level of internal consistency (Cronbach's α  = 0.96). The score for direct attitude ranged from 1 to 7.

Seven behavioural belief-based items have been used (e.g. Registering my consent to donate my eyes would make me feel good/bad); with each belief statement converted into seven corresponding outcome evaluations (e.g. Feeling better about myself is good/bad). Using recommended procedures [39], a unipolar response scale (1 to 7) was used for behavioural beliefs, with a bipolar response scale (−3 to +3) for outcome evaluations. The indirect measures of attitude have been obtained by multiplying the participant's perception about performing the behaviour (behavioural belief) with their corresponding evaluation of the consequences of performing the behaviour (outcome evaluation) [39]. The score for each indirect attitude item ranged from ±21. A composite score had then been obtained by summing all weighted behavioural beliefs [39] and ranged from ±147. *(See caveat below regarding scoring of belief-based measures using the multiplicative composite approach.).*

#### Subjective norm

Three items in total were included to assess direct subjective norms (e.g. Most people who are important to me think I should/should not register my consent to donate my eyes) and had a high level of internal consistency (Cronbach's α = 0.85). The score for direct subjective norm ranged from 1 to 7.

Seven normative belief-based items have been used (e.g. I think that my family would be likely/unlikely to want me to register my consent to donate my eyes); with each belief statement converted into seven corresponding motivational statements (e.g. I am likely/unlikely to do what my family thinks I should do). Using recommended procedures [39], a bipolar response scale (−3 to +3) was used for normative beliefs, with a unipolar response scale (1 to 7) for motivational outcomes. The indirect measures of subjective norm have been obtained by multiplying the participant's perception of the expectations of other referent individuals about performing the behaviour (normative belief) with their corresponding motivation to comply (motivational outcome) [39]. The score for each indirect subjective norm item ranged from ±21. A composite score had then been obtained by summing all weighted normative beliefs [39] and ranged from ±147. *(See caveat below regarding scoring of belief-based measures using the multiplicative composite approach.).*

#### Perceived behavioural control

Eight items in total were included to assess direct perceived behavioural control (e.g. I see myself as capable/incapable of registering consent to donate my eyes) and had an acceptable level of internal consistency (Cronbach's α  = 0.69). The score for direct perceived behavioural control ranged from 1 to 7.

Fifteen control belief-based items have been used (e.g. Having more information on how to register would make registering consent more likely/less likely); with each belief statement converted into 15 corresponding measures of control (e.g. Having more information on how to register would make registering consent much easier/much more difficult). Using recommended procedures [39], a unipolar response scale (1 to 7) was used for control beliefs, with a bipolar response scale (−3 to +3) for control outcomes. The indirect measures of perceived behavioural control have been obtained by multiplying the participant's perceived presence of factors that may influence or impede upon their ability (control belief) with their corresponding measure of control (control outcome) [39]. The score for each indirect perceived behavioural control item ranged from ±21. A composite score had then been obtained by summing all weighted control beliefs [39] and ranged from ±315. *(See caveat below regarding scoring of belief-based measures using the multiplicative composite approach.).*

#### Behavioural intention

Three items in total were included to assess behavioural intention (e.g. I agree/disagree that I intend to register my consent to donate my eyes) and had an excellent level of internal consistency (Cronbach's α  = 0.97). The score for behavioural intention ranged from 1 to 7.

#### The scoring of belief-based measures using the multiplicative composite approach

Despite the theory of planned behaviour being one of the most well-established and widely applied models of the socio-cognitive determinants in health and health-related behaviours [29], inconsistencies regarding the scoring methods employed for belief-based measures continue to exist [40]–[42]. Whilst it has been argued that belief-based measures should make use of a weighting process involving both a unipolar and bipolar scale [43], it has also been suggested that this may lead to statistically uninterpretable findings [40]–[42].

## Results

Participants were predominantly female (96.7%, n = 89), reflecting the gender base of the profession [44]. Age ranged from 18 to 46 years, with a mean of 24.0 years and a standard deviation of 5.6 years. The majority of participants (84.8%, n = 78) were under 30 years of age, with 13.1% (n = 12) aged between 30 and 39 years old and 2.2% (n = 2) aged 40 years or older. The majority had some form of religious affiliation (94.6%, n = 87), most frequently reported as either Protestant (58.7%, n = 54) or Catholic (35.9%, n = 33).

### Predicting intentions

To identify the factors influencing pre-registration nurses' intentions to personally register their consent, correlation and regression analyses were conducted. Means, standard deviations and correlations of intention among the theory of planned behavior constructs and beliefs have been shown in Table 1. All direct constructs and indirect measures of belief demonstrated a moderate to high level of correlation. For direct items, attitude exhibited the greatest degree of correlation with intention to register consent, followed by subjective norm and finally perceived behavioural control. For the belief-based measures, normative beliefs exhibited the greatest degree of correlation with intention, followed by behavioural beliefs and control beliefs.

**Table 1:** Means, standard deviations and correlations of intention with the theory of planned behaviour direct constructs (attitude, subjective norm and perceived behavioural control) and the respective underlying behavioural, normative and control beliefs.

Variable	1	2	3	4	5	6	7
(1) Intention	1						
(2) Attitude	0.91*	1					
(3) Subjective norm	0.67*	0.65*	1				
(4) Perceived behavioural control	0.60*	0.59*	0.44*	1			
(5) Behavioural beliefs^1^	0.45*	0.54*	0.42*	0.39*	1		
(6) Normative beliefs^1^	0.55*	0.57*	0.61*	0.31*	0.58*	1	
(7) Control beliefs^1^	0.44*	0.40*	0.43*	0.33*	0.37*	0.42*	1
							
Mean	3.72	4.71	3.73	5.85	73.89	31.80	−4.65
standard deviation	2.27	1.79	1.50	1.01	31.44	31.36	23.75

p<0.01.

1 Using a composite score.

In order to further explore the relative importance of the main independent variables in the prediction of intention, a hierarchical multiple regression analysis was conducted (Table 2). As religion was the only demographical item that correlated with intention, it was entered as an independent variable on Step 1 (F_1,85_  = 6.57, p = 0.012) and accounted for 7% (6% adjusted) of the variance in intention to register consent. The theory of planned behaviour constructs were added in Step 2 (F_4,82_  = 105.68, p<0.001) and accounted for 84% (83% adjusted) of the variance in intention to register consent (F change_3,82_  = 128.84, p<0.001). The standardised regression coefficients demonstrated that attitude was the strongest predictor of intention to register consent (β = 0.78, p<0.001) followed by subjective norm (β = 0.12, p = 0.045). However, the construct of perceived behavioural control failed to significantly predict intention to register consent (β = 0.08, p = 0.126).

**Table 2:** Hierarchical multiple regression of intention to register consent on religion (Step 1) and the theory of planned behaviour direct constructs (Step 2).

s	R	R^2^	Adjusted R^2^	F change	β
*Step 1*					
Religion	0.27	0.07	0.06*	6.57*	−0.268*
*Step 2*					
Religion					−0.004
Attitude	0.92	0.84	0.83**	128.84**	0.777**
Subjective norm					0.121*
Perceived behavioural control					0.084

* p<0.05; ** p<0.001.

### Relationship between intention and the indirect measures of belief

Table 3 highlights the relationship between intention to register consent and the beliefs that underpin the pre-registration nurse's attitude. It indicates that all seven beliefs found in the questionnaire correlate significantly with intention to register consent. Of those beliefs, helping to improve feelings of self-worth exhibited the greatest degree of correlation with intention, with family decision making showing the lowest degree of correlation with intention.

**Table 3:** Means and standard deviations of behavioural beliefs and Pearson's correlations (r) of behavioural beliefs with intention.

Beliefs	r	mean	sd
Make me feel better about myself	0.52*	7.61	7.52
Allow other people to think I am a better person	0.34*	3.93	5.98
Will set a good example for others to follow	0.28*	11.22	7.77
Will help to minimise family conflict	0.27*	5.30	6.32
Help to improve another person's life	0.25**	18.40	5.09
Give hope to someone in need	0.25**	17.83	5.14
Ensure that my family are in a better position to carry out my wishes	0.22**	9.60	7.88

* p<0.05; ** p<0.01.

Table 4 highlights the relationship between intention to register consent and the beliefs that underpin subjective norms. The results indicate that all seven beliefs correlate significantly with intention to register consent. Of those beliefs, pre-registration nurses expressed that their friends would most expect them to register consent, closely followed by family members. The lowest degree of correlation with intention to register consent was found with the expectations of charitable organisations that support people who need a corneal transplant.

**Table 4:** Means and standard deviations of normative beliefs and Pearson's correlations (r) of normative beliefs with intention.

Normative belief	r	mean	sd
My friends would want me to register	0.48*	−0.80	5.65
My family would want me to register	0.45*	−1.04	7.96
Health care professionals would want me to register	0.42*	5.11	6.36
The family of people who need a corneal transplant would want me to register	0.39*	9.77	5.90
People who need a corneal transplant would want me to register	0.38*	9.96	6.06
Religious groups would want me to register	0.33*	−0.08	5.20
Charitable organisations that support people who need a corneal transplant would want me to register	0.32*	8.89	5.83

* p<0.01.

Table 5 highlights the relationship between intention to register consent and the beliefs that underpin the pre-registration nurse's perceived behavioural control. The results indicate that of the 15 beliefs found in the questionnaire, three beliefs did not correlate significantly with intention to register consent. These included: body appearance; issues of mortality; and the existence of superstitions. For the beliefs that did correlate significantly, it is apparent that certain factors appear to encourage pre-registration nurses to personally register their consent. These include: improving access to information on how to register; increasing awareness of what eye donation involves; facilitating the process of registration at more convenient places; and raising awareness about the benefits of donation. Barriers to registering consent included: fears concerning other people looking through their eyes; issues of identity and impersonation; a general objection to ‘not having eyes’; and concerns that eye donation would result in a delay in burial.

**Table 5:** Means and standard deviations of control beliefs and Pearson's correlations (r) of control beliefs with intention.

Beliefs	r	mean	sd
Having more information on how to register	0.56*	4.18	9.68
Being more aware of what eye donation involves	0.51*	4.73	9.77
Being able to register at more convenient places	0.47*	4.16	8.07
Being more knowledgeable about the benefits of donation	0.46*	3.98	9.83
Having more opportunity to discuss donation with my family	0.43*	2.26	6.68
The thought of someone else looking through my eyes	0.42*	−2.07	6.95
The thought of someone else assuming my identity	0.40*	−2.70	6.81
Knowing that my decision to register would cause less distress to my family	0.35*	3.73	8.69
Knowing that my family would approve of my decision to register	0.34*	3.85	7.00
Knowing whether religious groups support organ donation	0.29*	0.10	5.14
The thought of not having my eyes	0.28*	−2.60	4.93
A possible delay in my burial	0.26**	−2.64	4.49
The appearance of my body after death without my eyes	0.18	−2.28	3.36
Having to think about death	0.18	−2.14	4.85
The thought of registration bringing me bad luck	0.13	−2.88	5.65

* p<0.05; ** p<0.01.

## Discussion

During its existence the theory of planned behaviour has been applied to a wide range of health and health-related behaviours, including that of organ donation [5]–[7], [9]–[10], [37], [45]. The results from these studies demonstrate that the theory of planned behaviour has been successful in explaining between 51% and 74% of the variance in intention to register as an organ donor [5]–[7], [9]–[10], [37], [45]. The findings obtained from this investigation provide further support for the predictive ability of the theory of planned behaviour as it has been capable of accounting for 84% (83% adjusted) of the variance in intention to register consent for corneal donation in this sample of pre-registered nurses (Table 2). What is striking is that the findings obtained are somewhat higher than the variance explained in previous studies [5]–[7], [9]–[10], [37], [45]. This higher level of variance is most likely to be the result of using a target population that has more altruistic tendencies (based on choice of profession) than that of the general population [46].

In relation to the constructs found in the theory of planned behaviour, attitude was found to be the strongest predictor of intention to register consent (Table 2). This is consistent with results from previous research that confirmed attitude as being the most significant predictor of intention to register consent for those considering organ donation in general [5]–[7], [10]. However, three groups found the construct of attitude to be the weakest predictor of intention [9], [37], [45]. Subjective norm was found to be the second strongest predictor of intention to register consent (Table 2) and is consistent with results from previous research [10], [37], [45]. Other studies have found the predictive ability of subjective norm to be either lower [5]–7 or higher [9] than that found in this investigation. In contrast to the predictive power of attitude and subjective norm, perceived behavioural control failed to significantly predict intention to register consent to corneal donation (Table 2). This lack of predictive ability is consistent with results from previous research that found registering consent to be within volitional control [10]. However, it is important to note that most other studies did find perceived behavioural control to have a significant role to play in helping predict intentions to register consent [6]–[7], [9], [37], [45].

The results also indicate that the majority of beliefs that underpin the constructs found in the theory of planned behaviour correlate significantly with intention to register consent. For instance, a positive association between registering consent and the benefits of improved self-worth and self-image has been confirmed in this investigation. This is not a surprising result considering that it has been shown that participation in a voluntary and altruistic act can positively impact upon the perception of an individual regarding his/her own character [47]–[48]. With regards to the beliefs that underpin subjective norm, the findings highlight those people who are considered influential in helping to make a well-informed decision. It is also apparent from the results that certain factors may encourage registration of consent. Therefore it would be important for awareness campaigns to be implemented and directed exclusively towards health professionals. This would be in a bid to ensure appropriate levels of knowledge are maintained on issues surrounding the benefits of donation while informing audiences about the process of eye donation in a manner that will be conducive to donation. This is an important aspect that needs to be considered, as it has been shown that the removal of certain barriers will positively impact upon the implementation of organ donation-related behaviours [49].

The results generated by this sample demonstrate merit in the need for further research to be conducted within this important area particularly as it has been recognised that many previous interventions have been ineffective at eliciting behaviour change [50] due to a lack of theoretical underpinning [51].

Further work needs to consider larger samples of groups such as nurses who are involved in organ donation and registration of potential donors. Future investigations comparing results from nurses originating and residing in different regions of the UK and in different countries are warranted. Longitudinal studies on the same cohort of individuals looking at questionnaires taken at pre-registration, early in post-registration and at regular intervals during their established careers would offer important insights into whether attitudes, perceptions, salient beliefs and behaviours alter with experience. In addition to nurses, attitudes, beliefs and perceptions of medical doctors and other health-care practitioners who deal with organ donation, as well as those of broader based cohorts from the general population, and indeed from a range of populations in different countries, need to be probed. This may indicate why wide variations in organ donation rates are found when making inter-nation comparisons [4].

This study utilised a convenience sample and it is recognised that the use of convenience sampling to recruit participants limits the ability to make generalisations about the total population from which the sample was chosen. However, the sample of future nurses was selected because a) it is nurses who predominantly deal with issues of organ donation and the recruitment of donors and b) a student population is most likely to provide responses that are as yet unaffected by experiences in professional practice and therefore will reflect the underlying beliefs of the individual. The sample was also biased towards females, which is indicative of the gender balance of the nursing profession [44]. Nevertheless, data generated by this convenience sample does provide valuable information about behaviour that may underlie corneal donation in a group of trainee allied health professionals.

Whilst the current study has adopted the method endorsed by Ajzen [30], the method for assessing expectancy-value and the inconsistencies that may arise with regard to scoring of belief-based measures has been challenged [40]–[42] and labelled the ‘expectancy-value muddle’. In essence, the measurement of beliefs with different forms of item scaling (uni- or bipolar) coupled with multiplicative composite of beliefs and evaluations of outcome renders the computation statistically lacking in interpretation [42]. Four alternative measures to resolve this have been suggested but all have either practical or conceptual limitations [42]. Recently, Newton et al have proposed a solution using dimensional salience [42]. This requires participants to choose a small number of salient beliefs from a list and, assuming that personally salient beliefs have a greater likelihood of eventuating than non-salient beliefs [52], these beliefs could be used to denote the expectancy component. The assumption was tested and found to be satisfied, indicating that the summation of a participant's value ratings of his/her salient beliefs could replace the multiplicative composite [42].

The methodology required for application of dimensional salience was not used in this study; belief-based items were obtained through an elicitation study. Whilst this study offers conceptual insights into the attitudes of a relatively homogenous cohort of trainee nurses; further investigations, designed in accordance with the dimensional salience approach [42] will provide additional statistical information that can offer better predictive capacity of attitude and intention.

### Conclusions

The present survey findings demonstrate that the theory of planned behaviour continues to provide a benchmark against which to investigate the socio-cognitive determinants of behaviour. More specifically, the theory of planned behaviour has allowed an understanding of the factors that influence the intention of pre-registered nurses based in Northern Ireland to register consent to donate their corneal tissue. It has been shown that attitude is the strongest predictor of intention to register consent, with subjective norm being ranked second and perceived behavioural control lacking any predictive ability. This adds to the body of knowledge about potential interventions that may alter donation-related behaviour.
